# Evaluation of vessel density in disorganization of retinal inner layers after resolved diabetic macular edema using optical coherence tomography angiography

**DOI:** 10.1371/journal.pone.0244789

**Published:** 2021-01-12

**Authors:** Gilda Cennamo, Daniela Montorio, Federica Fossataro, Claudia Fossataro, Fausto Tranfa

**Affiliations:** 1 Eye Clinic, Public Health Department, University of Naples “Federico II”, Naples, Italy; 2 Department of Neurosciences, Reproductive Sciences and Dentistry, University of Naples “Federico II”, Naples, Italy; University Hospitals Cleveland, UNITED STATES

## Abstract

**Purpose:**

To evaluate the retinal vessel density (VD) in the macular region and the foveal avascular zone (FAZ) area using optical coherence tomography angiography (OCTA) in patients with and without disorganization of retinal inner layers (DRILs) after resolution of diabetic macular edema.

**Methods:**

Thirty-seven eyes of 37 DRIL patients (mean age 63 ± 13.97 years), 30 eyes of 30 no DRIL patients and 35 eyes of 35 controls were enrolled in this study. We evaluated the VD in the macular region of superficial capillary plexus (SCP), deep capillary plexus (DCP) and FAZ area.

**Results:**

DRIL and no DRIL groups showed decreased VD in SCP and DCP (p<0.05) and a larger FAZ area (p<0.001) compared to controls. However, DRIL patients revealed a statistically significant reduction in VD of SCP (p = 0.041) and a greater FAZ area (p<0.001) with respect to no DRIL patients. We found a significant negative correlation between the VD of the foveal SCP (r = -0.414, p = 0.011), foveal DCP (r = -0.358, p = 0.025) and best corrected visual acuity (BCVA) in DRIL group. Moreover there was a significant positive correlation between the FAZ area (r = 0.425, p = 0.034) and BCVA. Therefore, in presence of DRILs lower VD values of SCP and DCP and a larger FAZ area correlated with a worse visual acuity. In no DRILs group, there was a significant negative correlation between the VD of the foveal DCP and BCVA.

**Conclusion:**

OCTA highlights the role of retinal vascular ischemia in the pathogenesis of DRILs. This parameter could represent an important functional predictive factor in diabetic patients.

## Introduction

Diabetic macular edema (DME) represents the main cause of visual loss in diabetic retinopathy [1] due to presence of intra-retinal and subretinal fluid in macular region [2–4].

Spectral Domain Optical Coherence Tomography (SD-OCT) allows to evaluate the architecture of the retinal layers identifying the disorganization of the retinal inner layers (DRILs) that consists of absence of identifiable boundaries between the ganglion cell-inner plexiform layer complex, the inner nuclear layer and the outer plexiform layer [5, 6].

Previous studies reported the close correlation in diabetic retinopathy between DRILs and macular capillary non-perfusion, detected by Fluorescein Angiography (FA). Furthermore, the presence of DRILs was associated with reduction of visual acuity in patients with DME or with resolved macular edema [7–11].

The evaluation of macular ischemia by FA, in presence of DRILs, did not allow a detailed evaluation of the superficial capillary plexus (SCP) and deep capillary plexus (DCP) in macular region [12–14].

The introduction of the OCT angiography (OCTA), a highly sensitive imaging technique, provided a quantitative assessment of the retinal microvasculature changes in patients with diabetic retinopathy [15–18].

The aim of this study was to analyze, using OCTA, the vessel density (VD) of SCP and DCP in macular region and Foveal Avascular Zone (FAZ) area as well as the correlation between these OCTA parameters and best corrected visual acuity (BCVA) in patients with and without DRILs after resolved DME.

## Materials and methods

### Subjects

In this retrospective study, from 95 patients with type 2 diabetes mellitus and ocular history of DME, a total of 67 eyes of 67 patients with resolved DME after anti-vascular endothelial growth factor injections were enrolled from January 2018 to July 2019 at the Eye Clinic of the University of Naples “Federico II”.

Each patient underwent BCVA evaluation according to the Early Treatment of Diabetic Retinopathy Study (ETDRS) (the BCVA was converted into LogMAR scale for statistical calculations), slit-lamp biomicroscopy, fundus examination, SD-OCT (Spectralis + HRA; Heidelberg Engineering, Heidelberg, Germany) and OCTA (RTVue XR Avanti, Optovue, Inc., Freemont, California, USA).

DRIL was defined as disorganizations of the inner retinal layers and, more precisely, as the inability to identify by SD-OCT the well-known-delineated boundaries between the ganglion cell-inner plexiform layer, inner nuclear layer and outer plexiform layer within the central 1500 μm region [7, 8, 10]. Patients showing resolved DME and DRIL represented the DRIL group; patients with resolved DME and without alterations in architecture of the retinal inner layers on SD-OCT B scans represented the no DRIL group.

After a retrospective review of SD-OCT images, we divided the patients into two groups: DRIL group that included 37 eyes of 37 patients (19 females, 18 males, mean age 63 ± 13.97 years) and no DRIL group including 30 eyes of 30 no patients (14 females, 16 males, mean age 62 ± 11.04).

In the DRIL group, 27 eyes showed non-proliferative diabetic retinopathy; while 10 eyes presented proliferative diabetic retinopathy. In no DRIL group, 20 eyes and 10 eyes showed non-proliferative and proliferative diabetic retinopathy, respectively.

Thirty-five eyes of 35 healthy subjects (16 females, 19 males, mean age 62 ± 10.72 years) served as control group, showing a normal ophthalmological evaluation, absence of vitreoretinal and vascular retinal diseases.

The evaluation of DRILs was performed by two masked examiners (FF, CF) and a senior expert (GC).

Exclusion criteria were the following: clinically significant lens opacities, low-quality of OCT and OCTA images, myopia greater than 6 diopters, presence of macular edema, subretinal or intraretinal fluid detected by SD-OCT with subfoveal thickness more than 300 μm [19], outer retinal layer disruption, history of choroidal neovascularization, uveitis, and intraocular surgery (including prior cataract surgery), absence of vitreoretinal, vascular retinal diseases (such as retinal vein or artery occlusion), and congenital eye disorders.

The study was approved by the Institutional Review Board of the University of Naples “Federico II” and all investigations adhered to the tenets of the Declaration of Helsinki. Written informed consents were obtained from the patients enrolled in the study.

### Optical coherence tomography angiography

The XR Avanti AngioVue OCTA (software ReVue version 2017.1.0.151, Optovue Inc., Fremont, CA, USA), is a device with a high speed of 70 000 axial scans per second that uses a light source of 840 nm and an axial resolution of 5 μm. This system is based on split-spectrum amplitude de-correlation algorithm (SSADA) which uses blood flow as intrinsic contrast. The flow is detected as a variation over time in a speckle pattern formed by the interference of light scattered by red blood cells and adjacent tissue structures [20].

The macular capillary network was visualized in scans centered on the fovea by performing a 6 x 6 mm scan. For each eye enrolled, the AngioAnalytic^TM^ software automatically calculated vessel density in different vascular networks of the retina SCP and DCP, analyzing the whole image, fovea and parafovea, according to the ETDRS classification of diabetic retinopathy [21].

The boundaries of superficial network extended from 3 microns below the internal limiting membrane to 15 microns below the inner plexiform layer (IPL). The deep capillary network extended from 15 to 70 microns below the IPL [22].

The software automatically calculated vessel density in whole scan area and in all sections of applied grid in different vascular networks of the retina. Vessel density (VD) was defined as the percentage area occupied by vessels in the analyzed region [23].

The projection artefact removal software was used in order to ensure correct visualization of the SCP and DCP [24].

Angiovue software automatically calculated the FAZ area in square millimetres over the 6 mm x 6 mm macular area in the full retinal plexus. The “non-flow” option was selected from a drop-down menu and the area was automatically selected when the FAZ area was clicked [25].

The images that presented a signal strength index less than 40, motion artefacts, incorrect segmentation, low centration and focus were not considered for the analysis.

### Statistical analysis

SPSS version 17.0 (SPSS Inc, Chicago, Ill, USA) was used for statistical analysis.

The Analysis of Variance (ANOVA) and chi-squared test were performed to evaluate the differences between groups in terms of age, BCVA and sex, respectively.

ANOVA with Bonferroni corrected for multiple comparisons was used to evaluate the differences in VD in each retinal vascular plexus, among DRIL, no DRIL groups and controls.

Pearson’s correlation was assessed between OCT angiography parameters (VD in SCP, DCP and FAZ area) and BCVA. A p value lower than < 0.05 was considered statistically significant.

## Results

DRIL patients, no DRIL patients and controls were not significantly different in terms of age (p = 0.922) and sex (p = 0.877). Conversely, BCVA was significantly different among the three groups (p<0.001). No significant association was found between the presence of DRIL or no DRIL and the stage of the diabetic retinopathy (p = 0.574). The demographic and clinical characteristics of the controls and patients was reported in Table 1.

**Table 1:** Demographic and clinical characteristics in controls, DRIL and No DRIL group.

	Controls	Dril Group	No Dril Group	AnovaP
**Eyes (n.)**	35	37	30	
**Female/Male**	16/19	19/18	14/16	0.877
**Age (years)**	62 ± 10.72	63 ± 13.97	62 ± 11.04	0.922
**BCVA, LogMar (Snellen)**	0.06 ± 0.08 (20/20)	0.71 ± 0.29 (20/100)	0.47 ± 1.65 (20/50)	**<0.001**
**DM duration (years)**	-	24 ± 11.13	23.6 ± 9.35	-
**Glycated hemoglobin level (%)**	-	7.2 ± 1.08	7.3 ± 1.09	-
** Nonproliferative diabetic retinopathy (eyes n.)**	-	27	20	
** Proliferative diabetic retinopathy (eyes n.)**	-	10	10	
**Treatment**	-	3.8 ± 1.5	3.2 ± 1.5	-
** Anti-VEGF injections (n.)**				

DM: Diabetes Mellitus; VEGF: Vascular Endothelial Growth Factor; BCVA: Best-Corrected Visual Acuity; LogMAR: logarithm of the minimum angle of resolution (Snellen equivalent inbrackets).

Data expressed as mean ± SD

Anova: Analysis of Variance with Bonferroni correction.

Bold p-values are significant (p <0.05).

**Fig 1:**
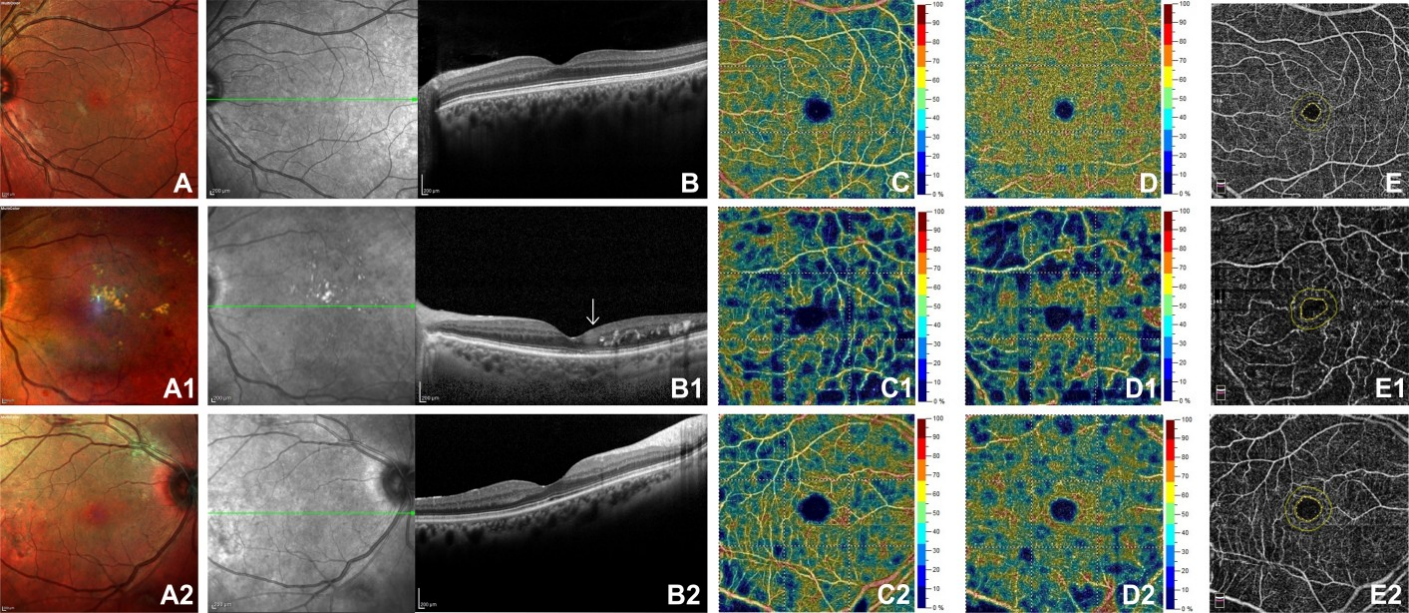
Multicolor image, Spectral Domain-Optical Coherence Tomography (SD-OCT), Optical Coherence Tomography Angiography (OCTA) in superficial capillary plexus (SCP), deep capillary plexus (DCP) and foveal avascular zone (FAZ) in a healthy control (top row, A-E), DRIL (disorganized inner retinal layer) patient (middle row, A1-E1) and no DRIL patient (bottom row, A2-E2). SD-OCT shows the area of DRIL (arrow) (B1) and well-delineated boundaries in inner retinal layers in no DRIL patient (B2) and in control (B). OCTA shows a reduction in vessel density in SCP, DCP and a greater FAZ area in DRIL (C1, D1, E1) and no DRIL (C2, D2, E2) compared control (C1, D1, E1).

Compared to controls, DRIL and no DRIL groups revealed a statistically significant reduction in VD of the SCP and DCP in the whole image, fovea and parafovea (p<0.001). Moreover, a statistically significant enlargement of the FAZ area was found in both groups with respect to controls (p<0.001). Lastly, DRIL patients showed significantly lower values of VD in SCP whole image (p = 0.041) and a greater FAZ area (p<0.001) compared to no DRILs patients (Fig 1).

The differences in VD and FAZ area values between the three groups are shown in Table 2.

**Table 2:** Differences in OCT angiography parameters among controls, Dril and No Dril groups.

	Controls	Dril Group	P^⁎^	No Dril Group	P^†^	P^‡^	Anova P
**Superficial Capillary Plexus (%)**							
***Whole image***	48.51 ± 4.32	41.15 ± 6.13	**<0.001**	44.17 ± 3.69	**0.002**	**0.041**	**<0.001**
***Fovea***	26.12 ± 8.68	16.08 ± 6.93	**<0.001**	17.26 ± 8.38	**<0.001**	1	**<0.001**
***Parafovea***	48.47 ± 7.83	42.14 ± 6.52	**<0.001**	43.48 ± 5.26	**0.010**	1	**<0.001**
**Deep Capillary Plexus (%)**							
***Whole image***	50.58 ± 4.37	42.16 ± 6.02	**<0.001**	43.72 ± 4.43	**<0.001**	0.634	**<0.001**
***Fovea***	37.93 ± 8.11	30.67 ± 9.13	**0.002**	32.20 ± 9.55	**0.034**	1	**0.002**
***Parafovea***	51.79 ± 5.49	46.43 ± 5.53	**<0.001**	47.94 ± 4.27	**0.011**	0.714	**<0.001**
**Foveal Avascular Zone (mm**^**2**^**)**	0.18 ± 0.06	0.65 ± 0.09	**<0.001**	0.33 ± 0.14	**<0.001**	**<0.001**	**<0.001**

Data expressed as mean ± SD.

Anova: Analysis of Variance with Bonferroni correction.

P*: Controls vs Dril Group.

P^†^: controls vs No Dril Group.

P^‡^: Dril Group vs No Dril Group.

Bold p-values are significant (p <0.05).

In the DRIL group we found a significant negative correlation between the VD of the foveal SCP (r = -0.414, p = 0.011), foveal DCP (r = -0.358, p = 0.025) and BCVA and a significant positive correlation between the FAZ area (r = 0.425, p = 0.034 showed) and BCVA. Therefore, lower VD values of SCP and DCP and larger FAZ area correlated with a worse visual acuity. In no DRILs group there was a significant negative correlation between the VD of the foveal DCP and BCVA (Table 3).

**Table 3:** Correlations between BCVA and OCTA parameters in Dril and No Dril Groups.

	BCVA	
	r	p
**DRIL GROUP**		
**Superficial Capillary Plexus**		
*Whole*	-0.207	0.219
*Fovea*	**-0.414**	**0.011**
*Parafovea*	-0.212	0.208
**Deep Capillary Plexus**		
*Whole*	-0.290	0.082
*Fovea*	**-0.358**	**0.025**
*Parafovea*	-0.214	0.203
**Foveal Avascular Zone**	**0.425**	**0.034**
**NO DRIL GROUP**		
**Superficial Capillary Plexus**		
*Whole*		
*Fovea*	-0.108	0.572
*Parafovea*	-0.064	0.736
	-0.017	0.928
**Deep Capillary Plexus**		
*Whole*	-0.207	0.273
*Fovea*	**-0.254**	**0.047**
*Parafovea*	-0.094	0.623
**Foveal Avascular Zone**	0.055	0.772

Pearson’s correlation p<0.05.

Bold p-values are significant (p <0.05).

## Discussion

To the best of our acknowledge, this is the first study evaluating the VD of the SCP and the DCP in macular region and the FAZ area in patients with DRILs and no DRILs after resolved DME.

Sun et al. supported, for the first time, the mechanical pathogenesis of DRILs due to macular edema that could cause the stretching of neuronal cells [6].

Whereas, Nicholson et al. proposed an ischemic hypothesis describing a significant association between the presence of DRILs and the macular non perfusion, evaluated by FA [7].

DRIL represents an OCT biomarker, able to predict the BCVA in patients with DME [10]; a study conducted by Radwan et al. showed the correlation between the length of DRIL and visual changes [25].

In our study, through a quantitative and detailed analysis of the retinal vascular networks by OCTA, we hypothesized that retinal ischemia plays a crucial role in the development of DRILs. Furthermore, we found a significant correlation between the presence of this OCT parameter and visual acuity loss.

Similar to the results reported by Moein et al., we found a significant increase in FAZ area in the DRIL group with respect to the no DRIL group and controls. Moreover, in patients with DRIL the larger FAZ area significantly correlated with lower BCVA while in the no DRIL group this relationship was not statistically significant [26].

In addition, DRIL and no DRIL patients showed a lower VD in both retinal vascular networks compared to controls, as also confirmed by Onishi et al. who found perfusion deficits in both SCP and DCP in DRILs areas showing a multilevel capillary non-perfusion in DRIL pathogenesis [27].

When comparing DRIL and no DRIL groups, we noticed that the first group showed a statistically significant reduction in VD of the SCP with respect to the second one. Thus, an altered perfusion of SCP, the main vascular network of the inner retinal structures [28], could contribute to the development of changes in the inner retinal architecture, supporting the vascular pathogenesis hypothesis.

In the correlation study, indeed, we reported a significant association between lower VD of SCP and DCP and decreased BCVA in the DRIL group, while only lower VD of DCP correlated with reduced BCVA in the no DRIL group, showing the possible influence of the vascular damage on vision loss in presence of DRILs.

We hypothesized that, in addition to the reduced perfusion of DCP mainly involved in diabetic retinopathy [29], lower VD of SCP in DRILs may cause an impairment of the metabolic activity in retinal inner layers, where the cells involved in synaptic transmission processes are located, contributing to the functional damage [30].

Possible limitations of this study are represented by its retrospective nature and by the relatively small number of eyes examined in each group.

In conclusion, our findings confirmed that the retinal non-perfusion in macular region plays an important role in the pathogenesis of DRILs.

OCTA provides an objective and quantitative analysis of VD, useful to better understand the changes in retinal vascular network occurring in DRILs and could represent an important functional predictive tool for patients with resolved DME.
